# Optimization of Culture Media for *Trichoderma* Strains as a Sustainable Approach for Agriculture

**DOI:** 10.1007/s00284-026-04921-2

**Published:** 2026-04-29

**Authors:** Isabela de L. Valente, Luciana Luft, Giovani L. Zabot, Marcio A. Mazutti

**Affiliations:** 1 Department of Chemical Engineering, Federal University of Santa Maria (UFSM), 1000 Roraima Av., Camobi, Santa Maria, 97105-340 RS Brazil; 2 Laboratory of Agroindustrial Process Engineering (LAPE), Federal University of Santa Maria (UFSM), 3013 Taufik Germano Rd, Cachoeira do Sul, 96503-205 Brazil

## Abstract

The intensive use of soil and the increased consumption of chemical fertilizers have risen over the years, causing soil degradation and impacting human health. The application of the fungus *Trichoderma* spp. has expanded exponentially and has been used to enhance soil fortification and detoxification of both soil and wastewater. Additionally, it has been applied in agricultural systems as a biocontrol agent against pathogens, to stimulate plant growth, and as a biological fertilizer to improve soil fertility. In this study, three *Trichoderma* strains (*T. harzianum* MMBF 58/09, *T. asperellum* URM 6997/160821, and *T. harzianum* IB 19/17) were cultivated using a culture medium designed through Plackett-Burman statistical design, followed by the characterization of the microorganisms to increase enzyme production, siderophore synthesis, and indole-3-acetic acid production. Significant enzymatic activity was observed, including chitinase (0.022 and 0.004 U·mL^− 1^), β-1,3-glucanase (0.199 and 0.007 U·mL^− 1^), and protease (1.45 and 1.40 U mL^− 1^), as well as propagules (9.67 × 10^7^ conidia·mL^− 1^, 2.56 × 10^7^ microsclerotia·mL^− 1^, and 1.79 × 10^7^ chlamydospores·mL^− 1^). Siderophore production reached 33.26%, and biomass was measured at 1.32 g·100 mL^− 1^. Some assays of the strains MMBF 58/09, URM 6997/160,821, and IB 19/17 demonstrated that these strains have promising potential to contribute to food safety and agricultural quality. Therefore, the observed responses emphasize the need for oriented microbial selection, supporting the development of targeted bioinputs within the framework of applied microbiology.

**Supplementary Information:**

The online version contains supplementary material available at 10.1007/s00284-026-04921-2.

## Introduction

In agricultural systems, including wheat, rice, maize, soybean, and potato crops, the demand for sustainable practices has increased, according to the FAO (Food and Agriculture Organization of the United Nations), due to current challenges such as the widespread use of agrochemicals that promote soil acidification and degradation. Additionally, inorganic-based fertilizers have emerged as an alternative to address nutritional deficiencies. However, they may also have negative impacts on soil biodiversity. Therefore, the next proposed approach is the use of biologically based fertilizers that improve soil fertility through organic and natural methods aimed at ecological enhancement. Soil microorganisms, such as bacteria and fungi, play a crucial role in nutrient cycling and ecosystem protection, which are vital for the proper functioning of agroecosystems. Some strains also serve as plant growth promoters and biocontrol agents. Among these microorganisms, *Trichoderma* has the potential to reduce dependence on mineral fertilizers, offering an ecologically sustainable approach that enhances soil health and decreases pesticide use [1, 2].

*Trichoderma* belongs to the order Hypocreales, the teleomorph of the phylum Ascomycota [3]. This filamentous fungus exhibits biocontrol mechanisms that include the production of metabolites with antimicrobial activity [4, 5]. It is characterized by rapid growth, antagonistic effects, and the ability to enhance nutrient availability and uptake, as well as the production of enzymes that degrade the cell wall. The metabolites produced can be either primary or secondary, the latter including volatile compounds [6]. *Trichoderma* can also induce plant resistance [7, 8], is commonly found in agricultural soils and forest environments, and demonstrates the ability to colonize both natural and artificial substrates rapidly. It contributes to plant growth through the production of phytohormones such as auxins (indole-3-acetic acid), in addition to promoting their transport and signaling, thereby benefiting root development and responses to stress. Furthermore, it is capable of mobilizing phosphorus through the production of extracellular phosphatases [9].

Fungi such as *T. harzianum* have shown positive effects on wheat yields by enhancing photosynthetic pigmentation and nutrient content (Fe, K, P, and Zn). *T. asperellum* demonstrated efficiency in seed germination, micronutrient levels, and lentil crop productivity when applied in association with nutrients such as Fe and Zn [9]. Siderophores are organic chelating compounds produced by microorganisms, considering that both plants and microorganisms require iron for growth and as an enzymatic cofactor to catalyze vital biological reactions [10]. The *Trichoderma* genus is also capable of producing proteins with enzymatic activity, including cellulases, chitinases, β-1,3-glucanases, proteases, and peroxidases, with a broad capacity to degrade recalcitrant compounds present in the soil. In addition, they produce hydrolytic enzymes and secondary metabolites with functions in antibiosis, competition for space and nutrients, thereby contributing to plant growth and enhancing resistance to both biotic and abiotic stresses [11].

Soil is a natural and living component involving multiple interactions between physicochemical and biological resources, with microbial communities playing a key role in soil functionality [7, 12]. The presence of *Trichoderma* in soil has been shown to affect pH regulation and the conversion of organic nutrients into forms accessible for plant uptake. Additionally, this influence provides energy to the soil microbiome and creates a favorable environment for its growth. In black pepper, *T. harzianum* altered the relative abundance of fungal species in the rhizosphere, leading to a predominance of beneficial fungi and a reduction in pathogens. Concerning the interaction of *Trichoderma* with soil bacterial populations in the presence and absence of the phytopathogen *Pythium ultimum*, it was observed that bacterial populations decreased without the pathogen, while in its presence, specific strains stimulated bacterial growth [1].

Microbial fermentation requires carbon and nitrogen sources for the growth and development of fungi such as *Trichoderma*, and consequently for the production of the desired product. Protein hydrolysates contain auxin precursors such as tryptophan and phenylalanine, signaling peptides, and metals that can contribute to plant growth [13]. In this context, the novelty of the present study lies in the systematic optimization of culture media for *Trichoderma* strains using a Plackett-Burman experimental design, coupled with an integrated evaluation of biomass and propagule (conidia, microsclerotia, and chlamydospores) production, enzymatic activities, siderophore synthesis, and indole-3-acetic acid (IAA) production. Unlike previous studies that typically focus on a single metabolite or strain, this work provides an assessment of multiple physiological and biotechnological traits under controlled fermentation conditions. This integrated approach offers new insights into how nutritional and physicochemical factors modulate *Trichoderma* performance, thereby contributing to the development of more efficient and sustainable microbial inputs for agricultural applications.

Therefore, this study aimed to evaluate and identify the most suitable culture medium to enhance enzymatic activities (chitinase, β-1,3-glucanase, and protease) and/or the production of siderophores and IAA, which are essential for plant growth and phytopathogen control, potentially applicable to agricultural systems, using the three *Trichoderma* strains obtained for this study.

## Materials and Methods

### Strains

**Fig. 1:**
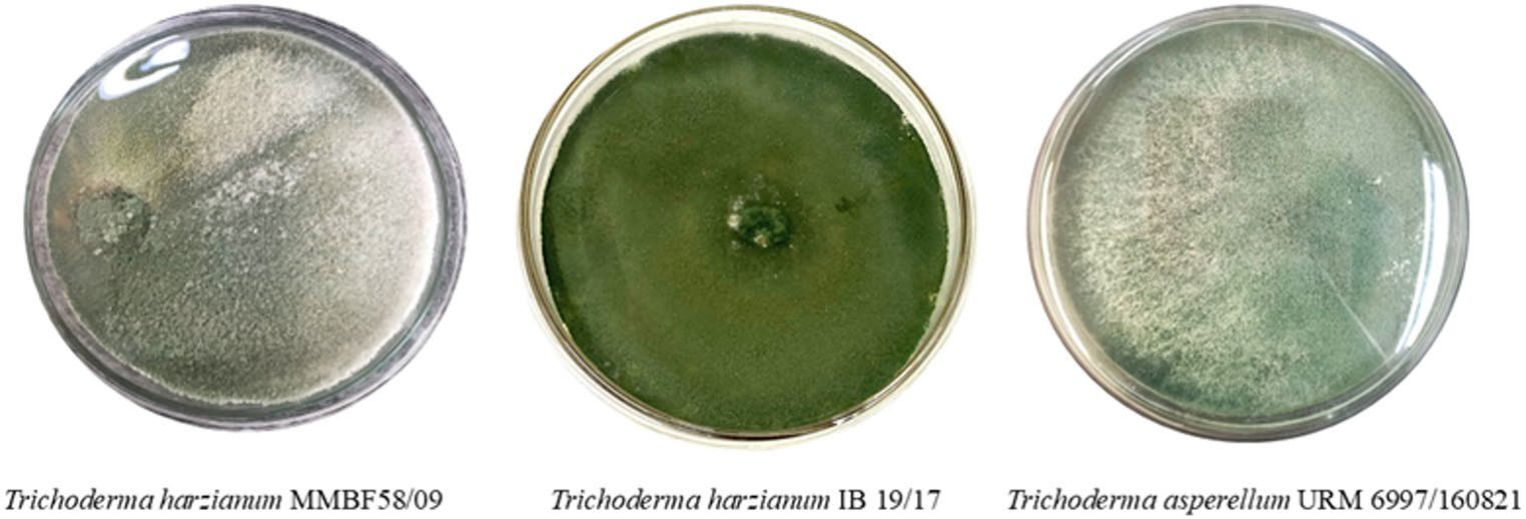
Colony morphology of the three *Trichoderma* strains used in this study grown on PDA medium: *T. harzianum* MMBF 58/09, *T. harzianum* IB 19/17, and *T. asperellum* URM 6997/160,821, showing characteristic mycelial growth and sporulation patterns under laboratory conditions

*Trichoderma* strains used in this study included *Trichoderma harzianum* MMBF 58/09, provided by the Biological Institute of São Paulo (Brazil), *Trichoderma asperellum* URM 6997/160,821, obtained from the Bioprocess Laboratory of the Federal University of Santa Maria (UFSM - Brazil), and *Trichoderma harzianum* IB 19/17, provided by the Biological Institute of Campinas (Brazil). The strains (Fig. 1) were cultivated on PDA medium (Potato Dextrose Agar EP/USP/BAM, KASVI) in triplicate for 7 days at 25 °C in an incubator under photoperiod conditions (SL 224, SOLAB, Brazil).

*Trichoderma* was selected for its non-toxic profile, endophytic behavior, ability to control pests and phytopathogens, capacity to produce enzymes and secondary metabolites, and to induce plant resistance. The MMBF 58/09 strain has been used in ongoing laboratory research, and the IB 19/17 strain was included for comparative purposes. The *T. asperellum* URM 6997/160,821 strain exhibited faster growth on PDA than *T. harzianum*. Therefore, all three strains were characterized individually to evaluate their production profiles, providing a basis for subsequent statistical analysis and optimization.

### Culture Conditions

The Plackett-Burman statistical design does not account for interactions between factors, being consistent in screening tests that identify the most favorable conditions [14]. The Plackett-Burman design is a factor-screening method used to identify variables with significant effects. However, it does not distinguish between main effects and interaction effects, as it compares only the differences between two levels of each factor. Consequently, factor interactions are not considered. Independent variables were calculated as the difference between the mean of the high level (+) and low level (-) divided by the number of trials. Central points were included to ensure reproducibility and the absence of curvature, as the mathematical model is considered linear [15].

In this study, the variables evaluated were glucose (Gl) (0, 10 and 15 g L^− 1^), sucrose (Su) (0, 5, 10 g L^− 1^), protein hydrolysate (HP) (2, 5 and 8 g L^− 1^), inorganic nitrogen (0, 1 and 3 g L^− 1^), and pH (4.5, 5.5, 6.5). They were tested over periods of 24 h, 48 h, 72 h, and 96 h, while maintaining basal medium (1 g L^− 1^ KCl, 1.05 g L^− 1^ K_2_HPO_4_, 0.36 g L^− 1^ KH_2_PO_4_, 0.6 g L^− 1^ MgSO_4_•7H_2_O, 0.05 g L^− 1^ C_2_H_5_NO_2_), temperature (28 °C), and agitation (150 rpm) constant. Response variables included counts of microbial structures (conidia, microsclerotia, and chlamydospores) and dry biomass. Fermentation was carried out by inoculating a 9 mm disc of the *Trichoderma* strain into 50 mL of culture medium. The Plackett-Burman design was applied for each strain (MMBF 58/09, URM 6997/160821, and IB 19/17), with inorganic nitrogen supplied as ammonium sulfate (AS). Experimental data were analyzed statistically by analysis of variance (ANOVA) with a significance level of 95% using Statistica^®^ 10 software.

Dry biomass was determined after fermentation of each fungus by filtration using a vacuum pump and 125 mm filter paper, pre-weighed. Following filtration, the filter paper with biomass was dried at 60 °C for 24 h. The dried samples were stored in a desiccator and weighed. Dry biomass weight was calculated as the difference between the filter paper before filtration and after drying. Cellular structures (conidia, microsclerotia, and chlamydospores) were quantified using a Neubauer chamber under an optical microscope.

#### Chemicals and Reagents

The animal protein hydrolysate sample (Faltechf4bs) contained 0% moisture, 80.8% protein, 10.9% fat, and 7.3% ash. Analyses were performed as follows: protein content by the Kjeldahl method (fc = 6.5) (AOAC, 981.10), fat by Soxhlet extraction (AOAC, 920.39), moisture (AOAC, 925.09), and ash content (AOAC, 942.05) determined gravimetrically in a forced-air convection oven and muffle furnace. Sucrose (Colombo brand, crystal type, Brazil), dextrose P.A. (Êxodo Científica brand, Brazil), and ammonium sulfate P.A. (Dinâmica brand, Brazil) were used.

### Characterization

#### Siderophore Production

Siderophores were quantified using the method of Schwyn and Neilands [16]. The fermented broth was centrifuged at 5,000 rpm, followed by the addition of CAS solution in a 1:1 ratio, and incubated for 60 min at room temperature (25 °C) in the dark. Absorbance was measured at 630 nm using a UV-VIS spectrophotometer (UV-2600, Shimadzu, Japan). A standard curve was prepared with EDTA-Na, and results were expressed as a percentage (%) based on the difference from the control.

#### IAA Production

IAA quantification was performed by filtering the fermentation medium, followed by assays using Salkowski reagent (1 mL of 0.5 M FeCl_3_•6H_2_O and 50 mL of H_2_SO_4_) in a 1:2 ratio, according to a modified method of Gordon and Weber [17], and incubated for 30 min at room temperature (25 °C). Absorbance was measured at 530 nm using a UV-VIS spectrophotometer (UV-2600, Shimadzu, Japan), and a standard curve was prepared with IAA [18].

### Enzymatic Assays

Chitinase activity was quantified through three parallel assays. First, 250 µL of 1% colloidal chitin solution as substrate, 200 µL of acetate buffer (50 mM, pH 5.0), and 50 µL of the sample were mixed. Second, 450 µL of acetate buffer (50 mM, pH 5.0) and 50 µL of the sample were combined as the sample blank. Third, 250 µL of 1% colloidal chitin solution and 250 µL of acetate buffer (50 mM, pH 5.0) were mixed as the substrate blank. All reactions were incubated at 37 °C for 30 min and stopped by placing the tubes in an ice bath. Subsequently, 500 µL of 3,5-dinitrosalicylic acid (DNS) solution was added, incubated at 95 °C for 5 min, and cooled on ice for 5 min. One enzyme unit was defined as the amount of enzyme required to release 1 µmol of substrate per minute, based on a standard curve prepared with N-acetyl-glucosamine. Absorbance was measured at 540 nm using a UV-VIS spectrophotometer (UV-2600, Shimadzu) [19].

The quantification of β-1,3-glucanase activity was carried out following the same procedure, with the difference that 1% laminarin solution was used as the substrate. The reactions were incubated at 45 °C for 30 min, and glucose was used to prepare the standard curve [19–21].

#### Protease Activity

Protease activity was quantified using casein as substrate at a concentration of 2% (w/v) in phosphate buffer (50 mM, pH 7.8). The assay consisted of mixing 0.5 mL of sample, 1.5 mL of substrate, and 1 mL of buffer, followed by incubation at 30 °C for 30 min. The reaction was stopped by adding 3 mL of trichloroacetic acid (TCA, 0.4 M) and cooling in an ice bath. Samples were centrifuged at 5000 rpm for 10 min, and absorbance was measured at 280 nm using a UV-VIS spectrophotometer (UV-2600, Shimadzu, Japan) [19, 22]. One unit of enzyme activity was defined as the amount of enzyme required to release 1 µg of tyrosine per milliliter per unit absorbance per minute [23].

## Results

### Biomass

The increase in biomass can be significant for a higher production of enzymes or volatile organic compounds favorable to biocontrol, as described for *Trichoderma* strains [24]. Accordingly, in this study, a statistical evaluation was carried out on the three *Trichoderma* strains, MMBF 58/09, URM 6997/160,821, and IB 19/17, as described in Table 1.

**Table 1:** Biomass (g·100 mL^− 1^), siderophore quantification expressed as Siderophore Units (%), and chitinase β-1,3-glucanase and protease activities (U mL^− 1^) at 24 h, 48 h, 72 h, and 96 h for the strains *T. harzianum* MMBF 58/09, *T. asperellum* URM 6997/160,821, and *T. harzianum* IB 19/17

	Biomass (g·100 mL^− 1^)											
	24 h			48 h			72 h			96 h		
Assay	MMBF 58/09	URM 6997/160,821	IB 19/17	MMBF 58/09	URM 6997/160,821	IB 19/17	MMBF 58/09	URM 6997/160,821	IB 19/17	MMBF 58/09	URM 6997/160,821	IB 19/17
1	0.53	0.71	0.50	0.83	1.00	0.98	0.84	0.99	0.94	0.81	0.72	1.01
2	0.32	0.71	0.33	0.63	0.81	0.75	0.67	1.16	0.78	0.71	1.20	0.87
4	0.53	0.62	0.67	0.81	1.02	0.77	0.77	1.01	1.04	0.98	1.32	1.04
5	0.32	0.61	0.38	0.59	0.91	0.79	0.57	1.08	0.85	0.75	1.19	0.72
6	0.70	0.47	0.52	0.93	0.95	0.89	1.12	1.00	0.98	1.08	1.15	1.00
	Siderophore Units (%)											
	24 h			48 h			72 h			96 h		
Assay	MMBF 58/09	URM 6997/160,821	IB 19/17	MMBF 58/09	URM 6997/160,821	IB 19/17	MMBF 58/09	URM 6997/160,821	IB 19/17	MMBF 58/09	URM 6997/160,821	IB 19/17
4	68.0	-	41.0	98.0	94.9	-	-	32.2	-	-	-	-
5	67.30	-	16.0	-	99.4	90.2	-	13.4	-	-	-	-
7	26.0	-	-	83.2	83.0	92.0	-	-	-	0.40	-	-
11	49.1	-	70.3	-	86.0	97.0	-	4.50	-	33.26	-	-
	Chitinase (U mL^− 1^)											
	24 h			48 h			72 h			96 h		
Assay	MMBF 58/09	URM 6997/160,821	IB 19/17	MMBF 58/09	URM 6997/160,821	IB 19/17	MMBF 58/09	URM 6997/160,821	IB 19/17	MMBF 58/09	URM 6997/160,821	IB 19/17
1	0.015	0.001	0.006	-	0.002	0.002	-	0.004	0.004	-	0.002	-
6	0.022	0.002	0.003	-	-	0.003	-	0.002	0.003	-	0.002	0.003
	β-1,3-glucanase ( U mL^− 1^)											
	24 h			48 h			72 h			96 h		
Assay	MMBF 58/09	URM 6997/160,821	IB 19/17	MMBF 58/09	URM 6997/160,821	IB 19/17	MMBF 58/09	URM 6997/160,821	IB 19/17	MMBF 58/09	URM 6997/160,821	IB 19/17
2	0.002	0.199	0.009	0.006	-	0.003	-	-	0.002	-	-	0.005
8	0.006	0.048	0.001	-	-	0.003	0.006	-	0.003	0.006	-	0.004
9	0.007	-	0.0001	-	-	0.004	0.006	-	0.005	0.007	-	0.009
10	0.007	-	0.002	0.004	-	-	-	-	0.003	-	-	0.006
	Protease (U mL^− 1^)											
	24 h			48 h			72 h			96 h		
Assay	MMBF 58/09	URM 6997/160,821	IB 19/17	MMBF 58/09	URM 6997/160,821	IB 19/17	MMBF 58/09	URM 6997/160,821	IB 19/17	MMBF 58/09	URM 6997/160,821	IB 19/17
1	1.23	1.18	0.93	0.68	0.47	0.67	0.54	0.47	0.47	0.39	0.94	0.74
3	0.89	0.18	1.45	1.07	0.18	0.75	0.60	0.18	0.67	0.53	0.28	0.98
6	1.33	1.01	0.89	1.02	0.67	1.40	0.81	0.67	1.15	0.89	1.34	1.47
7	1.14	0.55	1.14	0.92	0.85	0.94	0.76	0.85	0.90	0.83	0.74	0.48
8	1.25	0.86	0.63	0.64	0.49	0.98	0.62	0.49	0.66	0.07	1.34	-

**Fig. 2:**
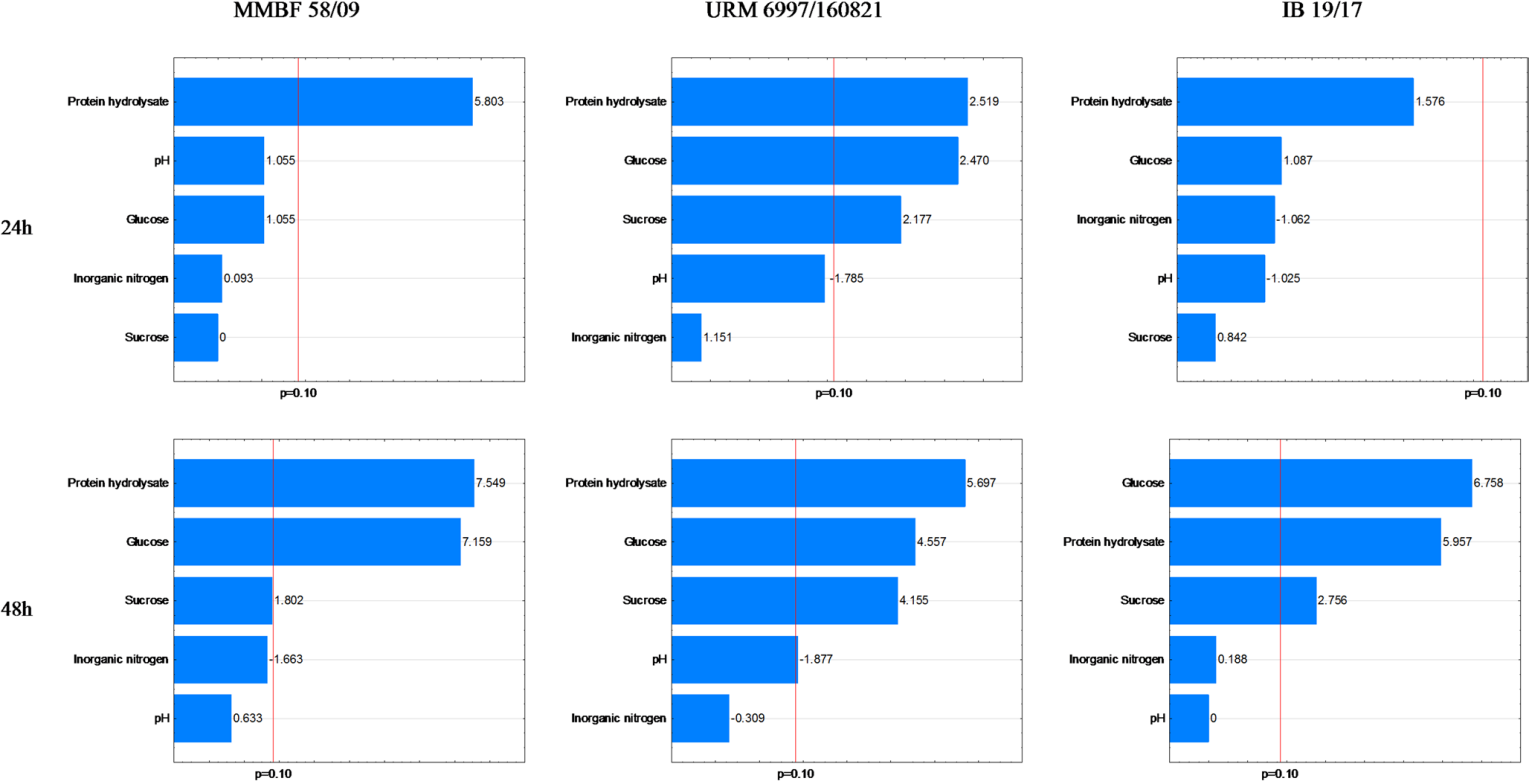
Pareto chart of biomass (g·100 mL^− 1^) for the strains MMBF 58/09, URM 6997/160,821, and IB 19/17 at 24 h, 48 h, 72 h, and 96 h

The significant values (*p* < 0.10) regarding the nitrogen source on biomass production indicated that AS concentrations were significant for strain IB 19/17 at 72 h and 96 h, whereas HP and Gl were not significant only at 24 h, Su at 48 h and 96 h, and pH at 96 h (Fig. 2). According to Table 1 and Supplementary Material (Table S1), the highest dry biomass values were obtained in assays 1 (96 h) with 1.01 g·100 mL^− 1^, 4 (72 and 96 h) with 1.04 g·100 mL^− 1^, and 6 (96 h) with 1.00 g·100 mL^− 1^. The carbon source Gl was significant at 48 h, 72 h, and 96 h in assays 1, 4, and 6, with a maximum of 15 g·L^− 1^, while Su reached the minimum (0 g·L^− 1^) in assays 1 and 4 and the maximum in assay 6 (10 g·L^− 1^). Similarly, pH was significant at 96 h, with a minimum of 4.5 in assays 1 and 4, and a maximum of 6.5 in assay 6.

According to Table 1, strain MMBF 58/09 achieved dry biomass values of 1.12 and 1.08 g·100 mL^− 1^ in assay 6 at 72 h and 96 h, respectively, under maximum HP (8 g·L^− 1^) and Su (10 g·L^− 1^) concentrations, and minimum Gl (5 g·L^− 1^). Significant effects (*p* < 0.10) were observed for HP at all times, for Gl at 48 h, 72 h, and 96 h, for Su at 72 h and 96 h, and for AS at 72 h (Fig. 2).

For strain URM 6997/160,821, assays 1, 2, 3, 4, 5, 6, and 7 showed dry biomass values above 1 g·100 mL^− 1^, ranging from 1.00 to 1.32 g·100 mL^− 1^ (Table 1). Significant effects (*p* < 0.10) were observed for HP, Gl, and Su across all times (Fig. 2). Under maximum HP and Gl conditions, as in assay 4, the highest biomass production was recorded at 96 h, reaching 1.32 g·100 mL^− 1^. When the pH for strain URM 6997/160,821 at 48 h and for strain IB 19/17 at 96 h, and the AS variable for strain MMBF 58/09 at 72 h reach their highest levels, negative effects indicate a decrease in biomass production, which aligns with assay 9 for all three strains.

### Propagule Count

**Fig. 3:**
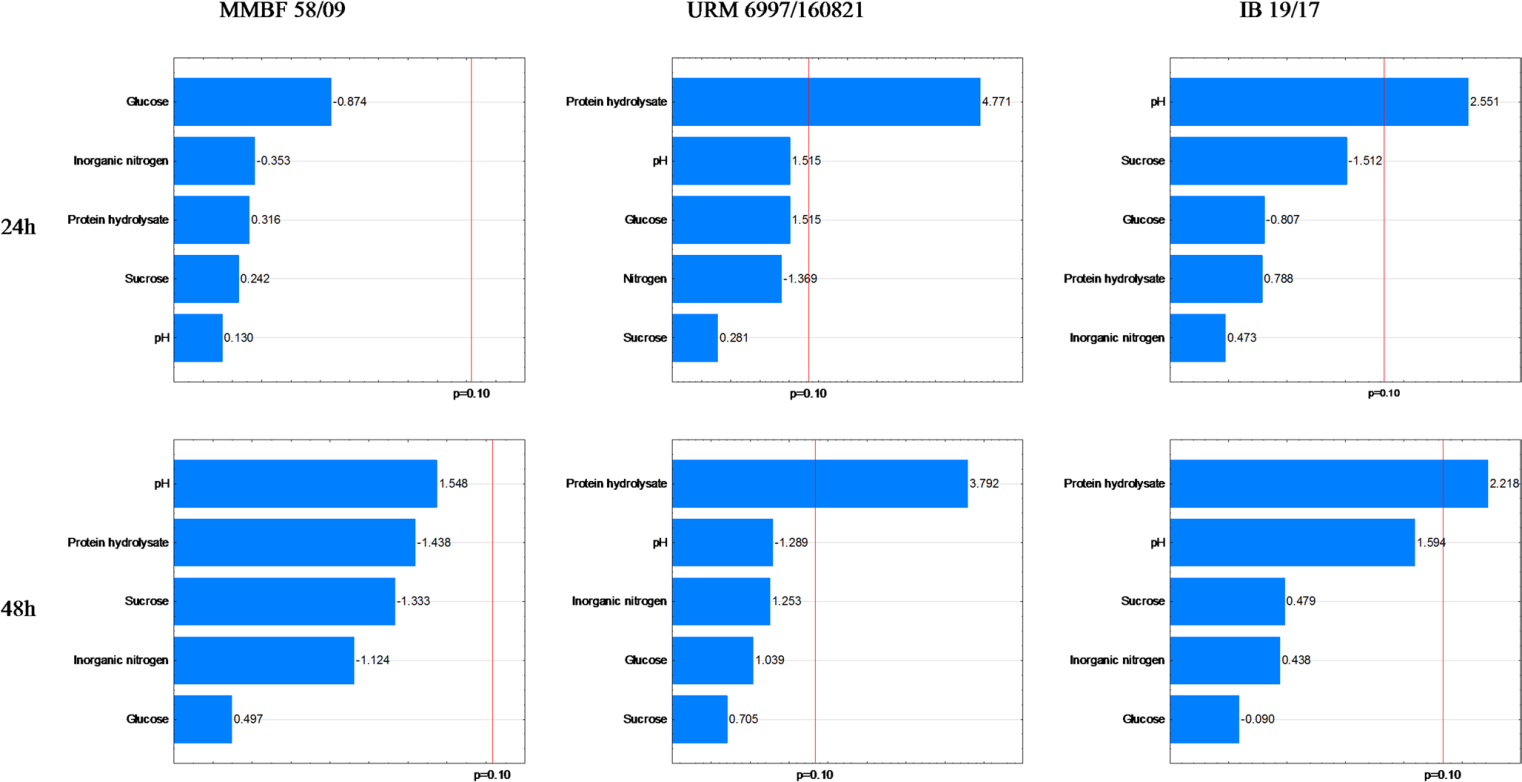
Pareto chart of conidial structures for the strains MMBF 58/09, URM 6997/160,821, and IB 19/17 at 24, 48, 72, and 96 h

**Fig. 4:**
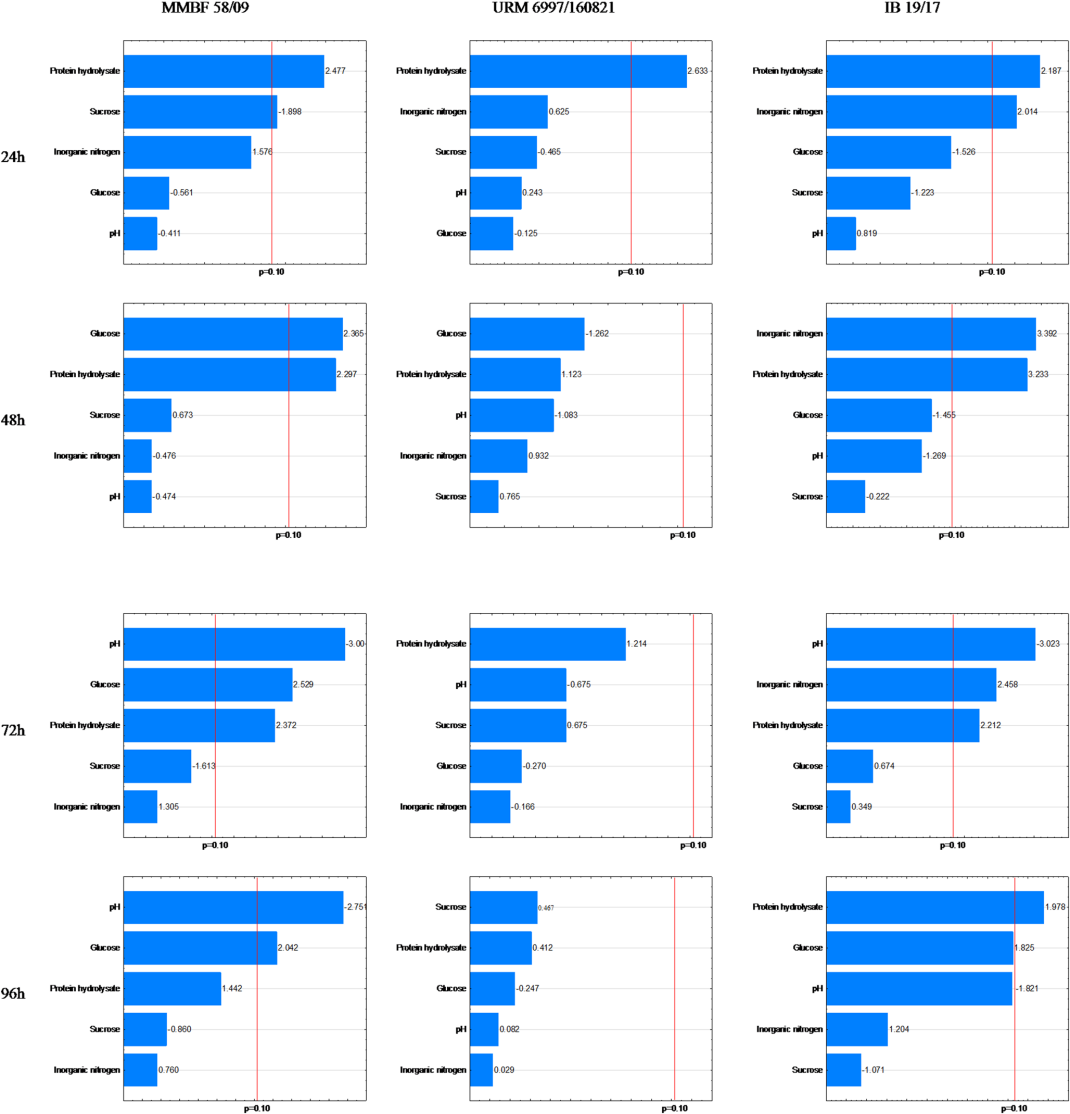
Pareto chart of microsclerotial structures for the strains MMBF 58/09, URM 6997/160,821, and IB 19/17 at 24, 48, 72, and 96 h

**Fig. 5:**
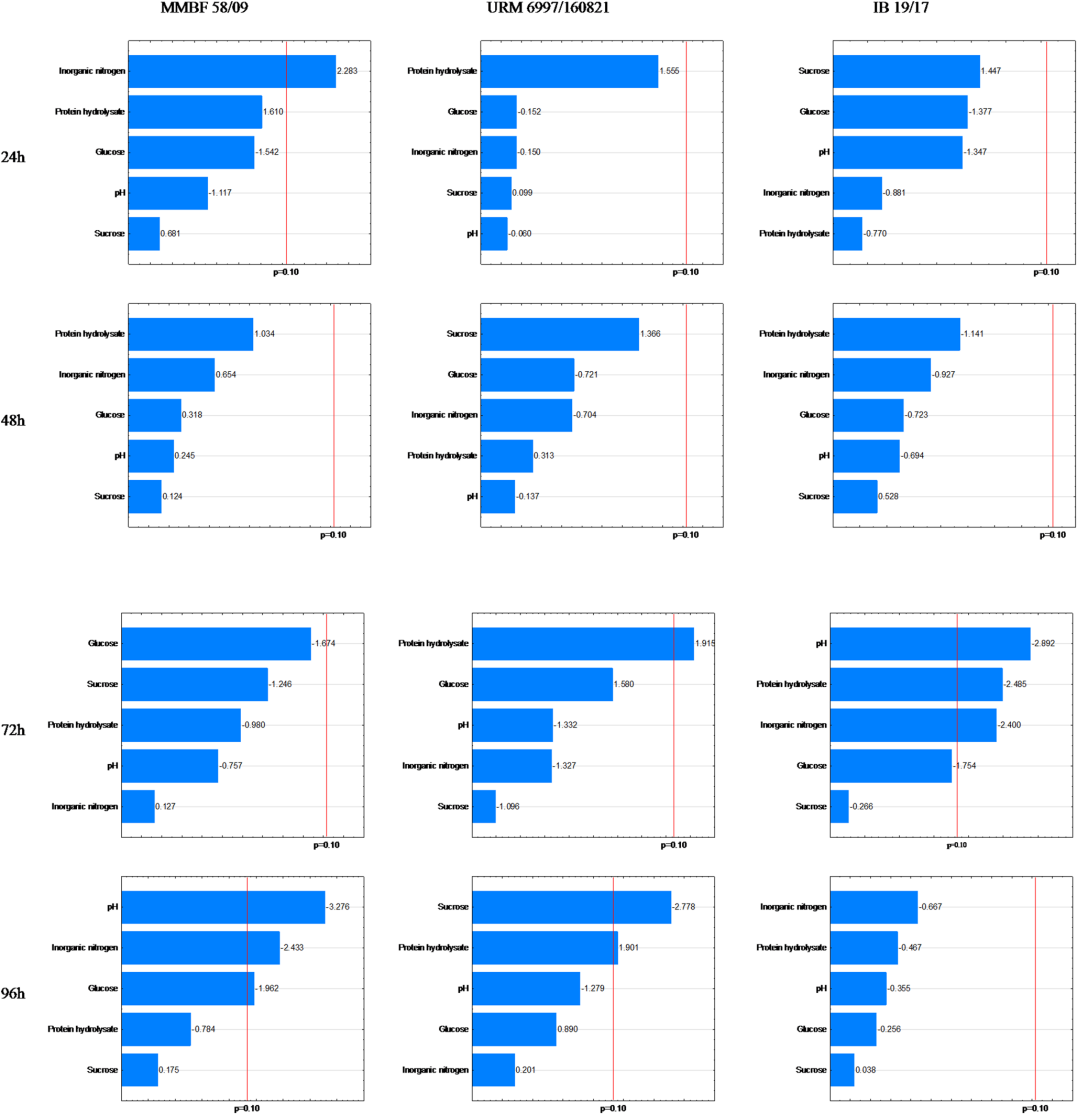
Pareto chart of chlamydospore structures for the strains MMBF 58/09, URM 6997/160,821, and IB 19/17 at 24, 48, 72, and 96 h

After counting the cellular structures of the MMBF 58/09 strain, as described in Table 2, the highest numbers of conidia were observed in assays 8, 9, and 10 at 72 h and in assays 3, 6, 8, 9, and 10 at 96 h. Regarding microsclerotia, the highest counts occurred in assays 1 and 8 at 24 h, assays 4 and 6 at 48 h, assay 4 at 72 h, and assays 1 and 4 at 96 h. For chlamydospores, assay 7 showed the highest count at 24 h, and assays 11 and 12 at 96 h. In the Plackett-Burman design (Figs. 3, 4 and 5), the quantification of conidia, microsclerotia, and chlamydospores showed statistical significance for the variables sucrose (Su) and pH at 72 h and pH at 96 h for conidia; HP and Su at 24 h, glucose (Gl) and HP at 48 h, pH, HP, and Gl at 72 h, and pH and Gl at 96 h for microsclerotia; and SA at 24 h and AS, pH, and Gl at 96 h for chlamydospores.

**Table 2:** Propagules per mL (conidia, microsclerotia, and chlamydospores) from fermentation with the MMBF 58/09 strain

	Propagules (mL^− 1^)											
	24 h			48 h			72 h			96 h		
Assay	Conidia (×10^5^)	Microsclerotia (×10^5^)	Chlamydospores (×10^5^)	Conidia (×10^5^)	Microsclerotia (×10^5^)	Chlamydospores (×10^5^)	Conidia (×10^5^)	Microsclerotia (×10^5^)	Chlamydospores (×10^5^)	Conidia (×10^5^)	Microsclerotia (×10^5^)	Chlamydospores (×10^5^)
1	4.50	112.0	27.0	4.50	91.8	22.8	22.0	136	30.8	23.5	112.0	58.5
2	7.00	49.3	40.0	14.0	48.8	53.5	11.0	45.0	23.0	8.50	26.3	24.5
3	6.00	11.8	15.0	36.0	9.00	43.8	17.5	24.0	10.8	127.0	12.3	6.25
4	6.00	72.3	5.75	8.00	168.0	27.0	15.5	242.0	45.5	66.0	133.0	7.00
5	1.50	1.25	1.00	12.5	19.3	13.8	16.5	29.8	37.0	58.0	52.3	5.50
6	17.0	11.3	4.25	6.00	265.0	17.5	27.0	0.75	13.3	585.0	1.00	36.3
7	14.0	59.8	94.0	20.5	48.8	11.8	36.5	39.5	32.5	27.5	33.0	41.5
8	10.5	212.0	45.8	35.0	3.25	77.8	599.0	0	63.3	321.0	1.00	30.0
9	14.5	21.3	27.3	55.5	0.75	9.25	758	0	84.8	434.0	0	1.00
10	6.00	0.25	0	249.0	0	0	718.0	0	1.5	967.0	0	0.50
11	11.5	10.5	4.00	31.0	25.8	15.8	13.0	15.8	62.3	17.0	43.5	104.0
12	9.00	17.0	3.25	21.5	12.8	7.25	7.00	9.50	115.0	10.5	15.0	107.0
13	23.5	47.3	7.00	53.0	65.5	43.5	25.5	28.3	39.0	11.0	12.8	16.3
14	14.0	60.0	2.5	52.0	14.5	32.3	22.5	18.8	129.0	11.5	6.5	20.8
15	23.5	70.5	33.8	69.5	35.8	79.3	14.5	13.5	96.0	16.5	9.75	47.3

The cellular structures of the URM 6997/160,821 strain, as described in Table 3, showed quantification of conidia in assay 6 at 24 h, assays 2 and 4 at 48 h, and assays 1 and 12 at 96 h. For microsclerotia, the highest counts occurred in assays 6, 7, 8, 14, and 15 at 24 h. Regarding chlamydospores, the highest counts were observed in assay 1 at 72 h and in assays 1, 4, and 8 at 96 h. Statistical significance was observed for HP at 24 h and 48 h, and pH at 96 h for conidia; HP at 24 h for microsclerotia; and HP at 72 h, and Su and AS at 96 h for chlamydospores (Figs. 3, 4 and 5).

**Table 3:** Propagules per mL (conidia, microsclerotia, and chlamydospores) from fermentation with the URM 6997/160,821 strain

Assay	24 h			48 h			72 h			96 h		
	Conidia (×10^5^)	Microsclerotia (×10^5^)	Chlamydospores (×10^5^)	Conidia (×10^5^)	Microsclerotia (×10^5^)	Chlamydospores (×10^5^)	Conidia (×10^5^)	Microsclerotia (×10^5^)	Chlamydospores (×10^5^)	Conidia (×10^5^)	Microsclerotia (×10^5^)	Chlamydospores (×10^5^)
1	11.0	12.5	3.50	10.0	2.25	2.5	8.00	2.75	64.5	19.0	0.25	54.0
2	5.50	0.25	0	12.5	0.75	10.5	12.5	0.50	10.5	13.5	1.00	3.25
3	16.0	1.00	52.8	10.5	0	23.8	7.50	0	11.8	1.50	0	0
4	10.0	15.0	49.5	13.5	0.75	5.50	4.50	0	10.5	10.0	0	29.5
5	3.00	0.25	0	3.00	0	4.00	4.00	0	0	3.00	0	2.75
6	12.5	23.0	3.00	8.50	0.75	0.25	14.5	4.00	9.50	13.5	3.00	1.75
7	9.00	28.0	1.50	11.5	27.5	1.75	13.0	8.00	0.75	12.0	3.50	1.00
8	7.50	26.5	3.25	11.5	3.25	1.00	15.5	0.75	5.75	7.50	0.50	25.8
9	3.00	6.5	0	0.50	2.75	0.50	1.00	0.25	0.25	8.50	2.25	16.3
10	9.50	8.25	1.00	1.50	0	0	1.50	0	0	1.50	0	0
11	0	1.00	8.00	1.00	1.00	6.75	2.50	0	0.25	1.00	0	7.00
12	2.50	0.50	1.50	2.00	1.75	5.75	10.5	1.25	2.75	34.5	0.25	4.50
13	5.50	16.5	1.50	10.0	9.50	3.50	15.0	5.75	17.5	9.00	5.5	24.8
14	6.50	21.3	1.00	11.5	10.5	3.00	8.50	3.25	7.00	10.5	4.25	7.50
15	6.00	26.0	8.00	12.5	11.0	12.0	14.0	8.00	9.75	11.0	6.00	15.3

The cellular structures of the IB 19/17 strain, described in Table 4, include conidia quantified in assays 8 and 10 at 24 h, assays 6 and 8 at 48 h, and assay 10 at 96 h. For microsclerotia, the highest counts were in assays 7 and 8 at 24 h, assays 4, 7, and 8 at 48 h, assays 4 and 7 at 72 h, and assay 4 at 96 h. Chlamydospores were highest in assays 11 and 12 at 72 h. Statistical significance was observed for pH at 24 h and 96 h, and HP at 48 h for conidia; HP and AS at 24 h, 48 h, and 72 h, pH at 72 h, and HP at 96 h for microsclerotia; and HP, AS, and pH at 72 h for chlamydospores (Figs. 3, 4 and 5).

**Table 4:** Propagules per mL (conidia, microsclerotia, and chlamydospores) from fermentation with the IB 19/17 strain

Assay	24 h			48 h			72 h			96 h		
	Conidia (×10^5^)	Microsclerotia (×10^5^)	Chlamydospores (×10^5^)	Conidia (×10^5^)	Microsclerotia (×10^5^)	Chlamydospores (×10^5^)	Conidia (×10^5^)	Microsclerotia (×10^5^)	Chlamydospores (×10^5^)	Conidia (×10^5^)	Microsclerotia (×10^5^)	Chlamydospores (×10^5^)
1	4.00	62.0	5.50	14.5	10.1	15.5	9.50	26.0	31.8	3.00	45.5	28.5
2	3.50	10.5	12.8	4.00	22.8	14.5	10.0	61.3	38.0	7.00	34.8	13.8
3	11.5	6.5	12.3	13.0	9.50	8.00	12.0	8.75	6.50	9.00	34.3	10.8
4	6.50	42.5	8.0	18.5	101.0	12.0	20.0	150.0	16.5	9.00	136.0	14.3
5	6.50	10.8	8.75	9.50	14.8	22.0	9.5	14.0	18.8	10.5	6.75	71.0
6	29.5	52.3	3.75	144.0	16.5	16.5	168.0	15.0	6.25	48.5	4.00	15.3
7	20.5	184.0	28.5	76.5	177.0	1.50	70.0	148.0	3.75	9.00	10.3	14.8
8	69.0	280.0	12.8	94.0	123.0	3.75	71.5	12.3	2.00	54.5	7.50	2.25
9	24.0	71.8	2.50	38.0	12.0	32.0	34.0	0.50	7.25	18.5	0.25	15.3
10	48.0	64.0	6.00	22.0	5.25	2.00	145.0	4.00	0.75	111.0	3.25	3.50
11	7.50	9.5	99.8	2.00	0	95.5	0	4.25	131.0	3.00	1.75	105.0
12	9.00	7.00	7.00	0.50	14.5	29.0	3.00	17.8	179.0	1.50	1.75	145.0
13	17.5	48.0	48.0	30.0	39.8	53.5	26.0	68.0	107.0	1.00	9.25	480.0
14	9.50	52.0	52.0	7.50	37.8	20.8	13.0	58.3	51.8	0.50	7.00	335.0
15	13.0	9.75	9.75	25.5	66.3	110.0	8.50	65.3	70.8	2.00	12.8	81.5

Negative effects show that for the MMBF 58/09 strain, biomass production drops when Su at 72 h (conidia), Su at 24 h, pH at 72 h and 96 h (microsclerotia), and Gl, AS, and pH at 96 h (chlamydospores) reach their highest levels. For the URM 6997/160,821 strain, high pH at 96 h (conidia) and Su at 96 h (chlamydospores) lowered production. For the IB 19/17 strain, high pH at 72 h (microsclerotia) and HP, AS, and pH at 72 h (chlamydospores) also decreased production. These findings align with assays 3, 5, 6, 8, and 9 across all three strains.

### Microbial Characterization

#### Quantification of Indole-3-acetic Acid (IAA)

The strain URM 6997/160,821 did not produce measurable IAA, while the quantification for strains MMBF 58/09 and IB 19/17 is presented in Supplementary Material (Table S3). IAA was not detected in all assays. The highest quantification was 0.80 mg mL^− 1^ for strain MMBF 58/09 and 0.10 mg mL^− 1^ for strain IB 19/17. The IAA test results were not statistically significant (*p* < 0.10) among the culture media components Gl, Su, HP, AS, and pH (data not shown).

#### Siderophore Production

The quantification of siderophores in this study was performed extracellularly, with the results presented in Table 1. Approximately 51.85% of the values were above 50% and were concentrated at 48 h for all three strains analyzed: MMBF 58/09, URM 6997/160,821, and IB 19/17. The highest siderophore percentage was observed for strain URM 6997/160,821, reaching 99.4% in assay 5, followed by 98% for MMBF 58/09 in assay 4 and 97% for IB 19/17 in assay 11. According to the Plackett-Burman design, only strain MMBF 58/09 showed statistical significance, with the highest quantified value of 33.26% in assay 11.

In the Plackett-Burman design, only the variables HP and AS showed significant values, with *p* < 0.05 for the strain MMBF 58/09 in Supplementary Material (Tables S4 and S5). Negative effects indicate that when these variables are at their highest level, siderophore production decreases, consistent with assay 7. Siderophore production values for the strains URM 6997/160,821 and IB 19/17 did not show statistical significance at *p* < 0.10.

### Enzymatic Activity

#### Chitinase

The results are presented in Table 1, with the highest chitinase quantification observed at 24 h for the strains MMBF 58/09 and URM 6997/160,821, and at 72 h for IB 19/17. The highest significant quantifications were 0.022 U mL^− 1^ in assay 6 for MMBF 58/09 and 0.004 U mL^− 1^ in assay 1 for IB 19/17. In the Plackett-Burman design, two times showed statistical significance. At 24 h, the variables Gl and HP presented significant values (*p* < 0.10) for the MMBF 58/09 strain, and Gl for the IB 19/17 strain in Supplementary Material (Tables S6 and S7). Chitinase production values for the URM 6997/160,821 strain did not show statistical significance at *p* < 0.10.

#### β-1,3-glucanase

The results of β-1,3-glucanase are presented in Table 1. The time showing the highest quantification of β-1,3-glucanase for the strains MMBF 58/09 and URM 6997/160,821 was 24 h, while for IB 19/17 it was 96 h. Considering only the statistically significant strains, the highest quantification was 0.007 U mL^− 1^ for strains MMBF 58/09 in assays 9 and 10 (24 h), 2 (48 h), 8 and 9 (72 h), and 9 (96 h), and 0.199 U mL^− 1^ for strain URM 6997/160,821 in assay 2.

In the Plackett-Burman design, in Supplementary Material (Tables S8 and S9), two strains showed statistical significance. For the MMBF 58/09 strain, the significant variables were pH (24 h), Su (48 h), Gl (72 h), and Su (96 h). For the URM 6997/160,821 strain, only the 24 h period showed statistical significance for the variables Su and pH, with *p* < 0.10. The β-1,3-glucanase quantification values for the IB 19/17 strain did not show statistical significance for *p* < 0.10. Negative effects indicate that when the variables are at their highest levels, β-1,3-glucanase production decreases. This is consistent with assays 1, 2, 4, 5, 6, and 10 at 72 h, and assays 2, 3, 5, 6, 7, and 11 at 96 h for the MMBF 58/09 strain, where no enzyme production occurred, and assays 3, 5, 8, 9, and 10 at 24 h for the URM 6997/160,821 strain.

#### Protease

The results of protease are presented in Table 1, and the time with the highest protease quantification for strain MMBF 58/09 was assay 8 at 24 h, followed by assay 3 at 48 h. For strain URM 6997/160,821, the highest values were observed in assays 1 and 6 at 24 h, followed by assay 7 at 72 h. For strain IB 19/17, the highest quantification occurred in assay 3 at 24 h and assay 6 at 48 h. The maximum quantification was reported in assays 3 and 6, with 1.45 and 1.40 mg mL^− 1^ for strain IB 19/17, assays 1 and 6 with 1.18 and 1.01 U mL^− 1^ for strain URM 6997/160,821, and assays 8 and 1 with 1.25 and 1.23 U mL^− 1^ for strain MMBF 58/09, respectively.

In the Plackett-Burman design, Supplementary Material (Tables S10 and S11), all three studied strains showed statistical significance. For strain MMBF 58/09, the variables Gl and HP were significant at 24 h and 48 h, Gl, HP, and AS at 72 h, and HP at 96 h. For strain URM 6997/160,821, HP was significant at 24 h, 48 h, and 72 h. For strain IB 19/17, HP was significant at 24 h and 48 h, while Gl and AS were significant at 72 h, with significant values at *p* < 0.10. Protease quantification values for strain IB 19/17 with negative effects indicate that when the AS variable is at its highest level, protease production decreases, consistent with assays 2, 5, 8, and 9 at 96 h, with the latter two showing no enzyme production.

## Discussion

For the efficiency of biocontrol, there is generally a search for antagonistic microorganisms for the management of phytopathogens. Microorganisms isolated from roots or the rhizosphere of a specific crop can be more effective due to their higher adaptability in disease control. In this context, *Trichoderma* shows vast biotechnological potential, with its efficiency being related to the physical, chemical, and biological conditions of the soil [25].

The Plackett-Burman statistical experimental design enables the identification of the main effects on efficiency to provide a fermentative medium with favorable conditions for *Trichoderma* culture, considering factors with a reduced number of experimental trials [26]. The assays that presented the highest biomass production were assay 6 for MMBF 58/09 and assay 4 for URM 6997/160,821 and IB 19/17, with both conditions corresponding to a ratio of 15 g L^− 1^ glucose to 8 g L^− 1^ protein hydrolysate. It is worth noting that the difference in assay 4 lies solely in the strain used (URM 6997/160821 and IB 19/17), since all parameters were otherwise identical. For strain MMBF 58/09, in addition to AS and pH not showing statistical significance at *p* < 0.10, there was an increase of 10 g L^− 1^ of Su.

Although the strains belong to the same genus, *Trichoderma*, they differ in their growth capacity when exposed to the same carbon and nitrogen sources [27]. In biomass and lactic acid production by *Rhizopus oryzae*, when varying the nitrogen source using chicken feather protein hydrolysate (with a high protein and ash content, 55.8 g (100 g)^−1^ and 42.1 g (100 g)^−1^, respectively, and essential amino acids for microbial growth), yeast extract, and ammonium sulfate, with beet molasses as the carbon source, the highest lactic acid yield corresponded to the hydrolysate at an optimal concentration of 7 g L^− 1^. To achieve this optimal hydrolysate concentration, it was reported that the C/N ratio in the medium can significantly affect microbial growth [28].

A high number of conidia contributes to accelerated proliferation and biomass synthesis. However, after a certain time, nutrient scarcity occurs, leading to a consequent reduction in metabolic activity. Determining the appropriate number of conidia can achieve a balance between nutrient availability and biomass accumulation required for enzymatic production [29]. In fermentations with *T. harzianum*, biomass increased in accordance with higher carbon availability over time and decreased linearly as the carbon source was depleted, regardless of the C/N ratio [30]. Nevertheless, in this study, biomass showed statistical significance for Gl and HP from 48 h onward in strains MMBF 58/09, URM 6997/160,821, and IB 19/17, with a C/N ratio of 15/8 g L^− 1^.

Similarly, conidial production (conidia mL^− 1^) was observed in assays 6 and 10 (96 h) and 10 (72 h) for MMBF 58/09, 1 (96 h) and 6 (24 h) for URM 6997/160,821, and 6 (48 h and 96 h) and 10 (24 h and 96 h) for IB 19/17, with maximum glucose (15 g L^− 1^), minimum hydrolyzed protein (2 g L^− 1^), absence of ammonium sulfate (0 g L^− 1^), and varying amounts of hydrolyzed protein. However, the main significant factors for the assays described were generally pH, followed by hydrolyzed protein, with conidial counts ranging from 967 to 1.25 × 10⁵ conidia mL^− 1^.

Low-carbon sources, such as sucrose in cultivation media with a C/N ratio close to 5/1 (soy peptone), were ideal for the growth of *T. atroviride*, allowing faster germination and higher bioactivity. In contrast, higher carbohydrate concentrations may trigger catabolite repression and reduce conidial production [31]. Assays such as 4 and 8 in the three studied strains, with low sucrose and high hydrolyzed protein and ammonium sulfate concentrations, showed considerable conidial production in the individual strains. Among the strains, MMBF 58/09 showed the highest conidial quantification, followed by IB 19/17, while URM 6997/160,821 exhibited the lowest production.

Propagules of filamentous fungi in *T. atroviride* remain metabolically active for up to 10 weeks of maturation, which confers characteristics similar to those of chemoautotrophic microorganisms. Furthermore, the transition from vegetative mycelium to conidia involves the formation of fruiting structures. In this process, the conidia formed enter a dormant state (this dormant form being a characteristic of ascospore-forming fungi) after the maturation stage. In *T. reesei*, it was discovered that conidia accumulate transcripts of several hydrolytic enzymes during their maturation [32]. The ability of microorganisms to absorb amino acids supports their growth [33].

In this context, microorganisms first utilize preferential or primary nutrients, repressing the use of secondary sources. When primary nutrients are depleted, metabolism shifts to secondary nutrient sources. This catabolite repression effect has been observed in *Trichoderma* spp. grown in nutrient-rich media. Additionally, primary nitrogen sources promoted conidiation induced by light more strongly than secondary sources. Thus, conidial production in *T. atroviride* depends on the carbon source in coordination with light induction, mediated by the expression of the blr1 and blr2 genes.

In *T. asperellum* and *T. pleuroticola*, conidiation was triggered by nitrogen catabolite repression, which in turn induced photoconidiation [31]. In a study with *T. harzianum*, submerged conidia were produced by conidiogenous cells (phialides) attached to hyphae at early growth stages on the second day of fermentation in low-carbon medium. As nitrogen concentrations decreased and carbon concentrations increased, conidial production also increased (7 days, 3.9–9.7 × 10^4^ conidia mL^− 1^), accompanied by improved vegetative growth [30].

Fungal propagules exhibit physiological differences regarding their production, stability, and microbial activity. Although aerial mycelium produces conidia and can adapt to adverse environments, propagules obtained from solid-state fermentations are more costly and present challenges for scale-up [34]. Liquid fermentation allows high yields of propagules with efficiency and stability through *Trichoderma*, provided strict quality control is maintained. However, these propagules are often produced with low soil persistence and storage instability. Thus, fungal microsclerotia are preferred for soil application due to their resistance structures [30], offering greater storage stability and higher tolerance to desiccation. Studies report that microsclerotia formation occurs under appropriate nutritional and/or environmental conditions. For example, *T. harzianum* T-22 forms microsclerotia over a wide range of C/N ratios with a carbon source concentration of 36 g L^− 1^, while other studies using nutrient-rich media with a lower C/N ratio and no vitamin supplementation utilized *T. asperellum* [34].

The highest microsclerotia production (microsclerotia mL^− 1^) for strain MMBF 58/09 was observed in assays 6 (48 h) and 4 (72 h and 96 h), for URM 6997/160,821 in assay 7 (24 h), and for IB 19/17 in assay 4 (72 h and 96 h), considering maximum glucose and minimum ammonium sulfate for assay 4. Both glucose and hydrolyzed protein were significant for MMBF 58/09, while only hydrolyzed protein was significant in assay 7, and hydrolyzed protein and ammonium sulfate in assay 1. Maximum hydrolyzed protein and ammonium sulfate were observed in URM 6997/160,821 and IB 19/17, with quantifications ranging from 0.25 to 28 × 10^5^ and 0.50–150 × 10^5^ microsclerotia mL^− 1^, respectively.

The formation of microsclerotia occurred in submerged media with *T. harzianum* under high carbon source conditions, and no submerged conidia were produced. In addition to the culture medium, the fermentation time impacted microsclerotia production, as growth began at 48 h, with the highest production (2.6–4.8 × 10^4^ microsclerotia mL^− 1^) and compactness observed on day 4 of fermentation, while more melanized and lower production was seen at day 7. Carbon sources (glucose and molasses) and nitrogen sources (soybean meal, cottonseed meal, yeast extract, corn steep liquor, and acid-hydrolyzed casein) supported submerged conidia production, whereas for microsclerotia, cottonseed meal combined with glucose yielded the highest production (C/N ratio 50/1), and molasses did not contribute to microsclerotia formation. The use of a 3-day submerged conidia pre-inoculum increased biomass and microsclerotia production compared to conidia taken from BDA plates [30].

Sucrose is a disaccharide that is easily assimilated compared to complex sugars, such as sorghum flour (used with *T. asperellum* strains), and is cheaper than glucose for use in submerged fermentations with filamentous fungi as biocontrol agents. Lower concentrations of carbon and nitrogen, in a 10:1 ratio (sucrose and autolyzed yeast), during seven days of submerged fermentation with *T. asperellum* yielded 2.5 × 10^4^ microsclerotia mL^− 1^ and 3.5 × 10^7^ CFU mL^− 1^ (colony-forming units representing total viable propagules) without vitamin supplementation [34].

Fermentations of *Trichoderma* spp. (*T. viride*, *T. hamatum*, and *T. harzianum*) in different media (corn mash molasses, sucrose nitrate, and glucose tartrate) resulted in the production of both conidia and chlamydospores while the pH fluctuations remained minimal. However, conidia production was more pronounced in static cultures, whereas chlamydospore formation predominated in liquid fermentations. Thus, propagule production depends on the isolate type, nutrient sources, and pH variation between 4 and 7 (Lnwts and Papavizas). Similarly, fermentations of different *T. viride* strains producing the antibiotic viridin exhibited microbial sporulation at intermediate to high pH, with high concentrations of glucose and nitrogen, and the strain showed enhanced sporulation when three or more trace elements, including iron, were added to the medium. Another strain performed better with intermediate glucose and low nitrogen concentrations [35].

LaeA is a global regulator whose loss leads to downregulation of aflRA, genetically identified in *Aspergillus nidulans*, and is known as a phylogenetically conserved methyltransferase in various filamentous fungi. It regulates secondary metabolism in *Trichoderma* spp., as well as conidia production, particularly in *T. longibrachiatum*. Propagule production, such as chlamydospores, which are thick-walled hyphal structures, occurs under adverse conditions, including significant pH changes, low temperature, and nutrient scarcity, as reported in *T. harzianum* [36]. Similarly, *T. asperellum* has been reported to enhance biomass production in water spinach (*Ipomoea aquatica*) [37].

Assays 1, 4, 8, 11, and 12 of the three studied strains showed significant values predominantly at the later fermentation stages (72 h and 96 h) with *p* < 0.10. The nutritional composition of the culture medium can affect fungal traits, including mycelial germination and conidia production. Therefore, careful optimization of carbon, nitrogen, and mineral sources is necessary to ensure optimal development, viability, and efficacy of the fungi selected as biological control agents. Achieving an appropriate carbon-to-nitrogen ratio is fundamental for high-quality conidia production [31].

The results of this study show that the strains MMBF 58/09, URM 6997/160,821, and IB 19/17 produced siderophores at all times, preferably at 48 h, with the highest production in assays 4, 5, and 11, respectively, although only strain MMBF 58/09 presented significant results at 96 h. Iron (Fe) is recognized as a critical factor affecting agricultural production due to its involvement in various essential biochemical and physiological processes in plants. Fe exists in two oxidation states, Fe²⁺ and Fe³⁺, which enable its roles in photosynthesis, respiration, nitrogen assimilation, and detoxification of reactive oxygen species. However, its concentration in soil is generally low and can be influenced by physical and chemical factors [38], and it is usually found in soluble, oxidized, or precipitated forms [39]. Siderophores are high-affinity ferric iron-chelating compounds produced by microorganisms and some plants under Fe deficiency, contributing to plant growth promotion and disease suppression [40].

Iron uptake in plants can be mediated by volatile organic compounds released by *Trichoderma*. For instance, the strains *T. harzianum* T78 and *T. asperellum* T34 promoted the expression of IRT1 and FRO2 in *Arabidopsis thaliana*. In soybean roots, *T. afroharzianum* T22 is capable of inducing the expression of citrate synthase (GmCs) and malate synthase (GmMs), which are responsible for the production of organic acids that lower soil pH and enhance Fe chelation following Fe deficiency, as soil acidification is a key mechanism for Fe solubilization. *Trichoderma* is also able to produce metabolites that function as siderophores, which are essential for fungal Fe homeostasis and related activities [41].

Enzymatic activity quantification revealed chitinase, β-1,3-glucanase, and protease production, with the highest chitinase activity observed in assays 6 (MMBF 58/09) and 1 (IB 19/17) at 24 h; β-1,3-glucanase in assays 2 (48 h), 8 (72 h), 9 (24 h and 96 h), and 10 (24 h) for MMBF 58/09, as well as assay 2 (URM 6997/160821) at 24 h; and protease in assay 6 for all three strains at 24 h (MMBF 58/09) and 96 h (URM 6997/160821 and IB 19/17). Regarding enzymatic activities, chitinases are responsible for hydrolyzing chitin, a linear polymer composed of N-acetyl-2-amino-deoxy-D-glucopyranose or N-acetylglucosamine units linked by β(1→4)-glycosidic bonds [29]. In a study cultivating *Trichoderma* isolates in a minimal synthetic buffered medium using 1% colloidal chitin as a carbon source, the isolates showed maximum activity equivalent to 62.12 pka mL^− 1^ (3.73 × 10^− 3^ mg mL^− 1^) of chitinase in *T. harzianum* and 9.94 nkat mL^− 1^ (5.96 × 10^− 1^ mg mL^− 1^) of β-1,3-glucanase in *T. viride* when 1% laminarin was used as the carbon source. These activities were analyzed with respect to pH and temperature: chitinase activity increased within a pH range of 3.0–6.0 and temperature of 15–30 °C, while β-1,3-glucanase activity varied within pH 3.0-5.5 and 15–25 °C. Another effect was observed when other carbon sources, such as glucose and sucrose, were used, which suppressed both chitinase and β-1,3-glucanase activities compared to colloidal chitin and laminarin, respectively. As a nitrogen source, ammonium nitrate resulted in the highest enzymatic activity [25].

In the present study, no supplementation with colloidal chitin or laminarin was used, and the enzymatic activity values were similar. Assay 6 showed the highest chitinase quantification at 24 h for the MMBF 58/09 strain, and assay 1 at 72 h for IB 19/17, both conducted at 28 °C with 15 g L^− 1^ glucose, without ammonium sulfate as the nitrogen source but with 8 g L^− 1^ of hydrolyzed protein (HP). Another study using solid-state fermentation with *Trichoderma* and colloidal chitin as the carbon source reported increased chitinase production compared to other sources, such as chitin powder or flakes, due to the colloidal conformation facilitating microbial metabolization. For nitrogen sources, yeast extract yielded the highest enzymatic activity compared to peptone, urea, corn steep liquor, ammonium chloride, and ammonium sulfate [29].

In a study on *T. harzianum* cultivation for β-1,3-glucanase production, an increase was observed at 24 h, with maximum activity reached at 96 h, and the optimal pH range was 4.0–6.0 [42]. The production of chitinase and β-1,3-glucanase is influenced by pH, with acidic conditions reported as critical for enzymatic production in *T. harzianum*, showing an optimum pH of 6. Another important factor is the carbon source; high enzymatic activity requires induction by fungal cell wall components [25–43], which contain linear ordered chitin and amorphous β-1,3-glucan [44]. In contrast, carbon catabolite repression can occur with sugars such as glucose and fructose. Another study reported that glucose can act as a catabolic repressor in enzyme production, while sucrose may be associated with lower protein synthesis [45]. Assays 2 (48 h), 8 (72 h), and 9 (24 h, 72 h, and 96 h) showed the highest β-1,3-glucanase activity for strain MMBF 58/09, and assay 2 at 24 h for strain URM 6997/160,821.

The determination of β-1,3-glucanase enzymatic activity depends on several factors, including the strain used, culture conditions, and the type of substrate/carbon source. In a liquid fermentation using TLE medium with *T. asperellum*, varying substrates such as glucose, starch, chitosan, chitin, laminarin, CWRS (cell wall of *Rhizoctonia solani*), and cellulose were tested for β-1,3-glucanase production. The highest enzyme activity was obtained with CWRS and starch, while the other polysaccharides showed lower activity, and no activity was detected with glucose as the carbon source. The activity levels varied according to the type of glycosidic linkage and the carbohydrate structure. Additionally, β-1,3-glucanase activity was observed in *T. harzianum* grown with the cell walls of phytopathogens such as *R. solani*, *Sclerotium rolfsii*, and *Pythium* spp. The optimal pH was in the range of 4.0–6.0, precisely 3.6, and the optimal temperature was 45 °C [44].

Among the sugars tested, the highest β-1,3-glucanase activity was observed with galactose, and the lowest with sucrose. For chitinase, the highest activity was observed with glucose and the lowest with maltose. Indirectly, *T. viride* exhibited maximum biomass production when ammonium sulfate was used as the nitrogen source [25]. In this study, significance for ammonium sulfate was observed at *p* < 0.10 in assay 2 (48 h) and 9 (96 h) for strain MMBF 58/09, and at 24 h for strain URM 6997/160,821, regarding β-1,3-glucanase activity, with the highest concentration in assay 2. Chitinase activity reached its maximum in assays 1 and 6 for strains IB 19/17 and MMBF 58/09, respectively. Ammonium sulfate did not show significance for strains MMBF 58/09 and URM 6997/160,821, only for IB 19/17 at 72 h, with maximum production observed in assay 6 for strain MMBF 58/09 and assay 4 for strains URM 6997/160,821 and IB 19/17, at the highest hydrolyzed protein (HP) concentration of 8 g L^− 1^. The addition of glucose to a fermentation medium for β-1,3-glucanase production may be advantageous when using *T. harzianum*, but for other species, enzyme synthesis can be inhibited in the presence of high levels of glucose or readily fermentable carbon sources [46].

IAA is a plant hormone that supports plant growth, similar to auxins, promoting root development during early plant stages. *Trichoderma* species are capable of producing the metabolite IAA [1]. Studies with *Trichoderma* in submerged fermentation using Tryptic Soy Broth reported IAA production ranging from 7.19 to 21.14 × 10^− 3^ mg mL^− 1^, which was increased by the addition of L-tryptophan [18]. Similarly, the addition of L-tryptophan in Potato Dextrose broth fermented with *Diaporthe terebinthifolli* resulted in a maximum IAA production of 121.21 × 10^− 3^ mg. mL^− 1^, and higher pH values can lead to elevated IAA production in *T. harzianum*. This effect may be due to alterations in protein conformation, causing enzyme denaturation and modifying the metabolite profile. A similar effect can occur with increased temperature, as temperatures between 30 and 35 °C in *Trichoderma* promote higher IAA production [47].

IAA production is not a universal trait among all *Trichoderma* species. Nevertheless, the genus can promote plant growth regardless of the environment from which strains are isolated. In fungi, IAA biosynthesis is tryptophan-dependent, and adding amino acids to the culture medium enhances its production. Also, factors such as pH, temperature, and carbon and nitrogen sources significantly influence IAA biosynthesis [48–49].

In this study, the assays showing the highest protease quantification and significance at *p* < 0.10 were assays 1 and 8 for strain MMBF 58/09, 1 and 6 for strain URM 6997/160,821 at 24 h, and 3 at 24 h and 6 at 48 h for strain IB 19/17. All three strains showed significance for hydrolyzed protein (HP). According to Metwally et al. [50], who used organic residues (fish waste) on onion seeds (*Allium cepa* L.) together with mycorrhizal fungi and *Trichoderma*, an increase in onion shoot growth was observed when co-inoculated with mycorrhizal fungi and *T. viride*, as well as enhanced root surface area and nutrient accessibility, more when the fungi were applied individually.

## Conclusion

The three *Trichoderma* strains exhibited distinct and complementary physiological responses when cultivated under optimized culture media conditions defined by the Plackett-Burman design. No single strain consistently outperformed the others across all evaluated parameters, including biomass production, propagule formation, enzymatic activities, siderophore synthesis, and IAA production. It highlights a clear dependent specialization, reflecting adaptive strategies that are characteristic of such a genus of fungi. While some strains showed superior biomass accumulation and propagule yields, others were more efficient in producing hydrolytic enzymes or siderophores, indicating that these traits are regulated independently and influenced by both genetic background and nutritional conditions.

From a microbiological and biotechnological perspective, these findings emphasize that the selection of *Trichoderma* strains for agricultural applications should be guided by specific functional objectives, such as enzyme production, nutrient mobilization, or propagule stability, rather than by the expectation of a universally superior strain. The optimized media identified in this study provide a valuable framework for enhancing targeted microbial functions, contributing to the rational development of sustainable bioinputs. Overall, this work advances the understanding of how closely related *Trichoderma* strains respond differentially to culture conditions, reinforcing the importance of comparative physiological analyses in applied microbiology and supporting the strategic use of microbial diversity in sustainable agricultural systems. It is important to consider that strict control of the C/N ratio in the medium is necessary, as it can affect microbial growth. Further studies are required to optimize the medium to enhance the quantification of the responses evaluated in this work.

## Electronic Supplementary Material

Below is the link to the electronic supplementary material.

Supplementary Material 1

## Data Availability

No datasets were generated or analysed during the current study.
