# Supplementary material for: Optimization of Culture Media for Trichoderma Strains as a Sustainable Approach for Agriculture
Source: Curr Microbiol. 2026 Apr 29;83(6):339. doi: 10.1007/s00284-026-04921-2 (PMC13128744; doi:10.1007/s00284-026-04921-2)
Supplement: Supplementary file 1 — Supplementary Material 1 [file 284_2026_4921_MOESM1_ESM.docx]

**Supplementary Material**

Table S1 Biomass values (g·100 mL^-1^) at 24 h, 48 h, 72 h, and 96 h for the strains *T. harzianum* MMBF 58/09, *T. asperellum* URM 6997/160821, and *T. harzianum* IB 19/17

|  | Biomass (g·100 mL^-1^) | | | | | | | | | | | |  |
| --- | --- | --- | --- | --- | --- | --- | --- | --- | --- | --- | --- | --- | --- |
|  | 24 h | | | 48 h | | | 72 h | | | 96 h | | |  |
| Assay | MMBF 58/09 | URM 6997/160821 | IB 19/17 | MMBF 58/09 | URM 6997/160821 | IB 19/17 | MMBF 58/09 | URM 6997/160821 | IB 19/17 | MMBF 58/09 | URM 6997/160821 | IB 19/17 | |
| 1 | 0.53 | 0.71 | 0.50 | 0.83 | 1.00 | 0.98 | 0.84 | 0.99 | 0.94 | 0.81 | 0.72 | 1.01 | |
| 2 | 0.32 | 0.71 | 0.33 | 0.63 | 0.81 | 0.75 | 0.67 | 1.16 | 0.78 | 0.71 | 1.20 | 0.87 | |
| 3 | 0.44 | 0.59 | 0.52 | 0.71 | 0.95 | 0.71 | 0.81 | 1.05 | 0.62 | 0.77 | 0.98 | 0.58 | |
| 4 | 0.53 | 0.62 | 0.67 | 0.81 | 1.02 | 0.77 | 0.77 | 1.01 | 1.04 | 0.98 | 1.32 | 1.04 | |
| 5 | 0.32 | 0.61 | 0.38 | 0.59 | 0.91 | 0.79 | 0.57 | 1.08 | 0.85 | 0.75 | 1.19 | 0.72 | |
| 6 | 0.70 | 0.47 | 0.52 | 0.93 | 0.95 | 0.89 | 1.12 | 1.00 | 0.98 | 1.08 | 1.15 | 1.00 | |
| 7 | 0.59 | 0.50 | 0.22 | 0.72 | 0.90 | 0.72 | 0.83 | 1.02 | 0.90 | 0.79 | 0.98 | 0.68 | |
| 8 | 0.56 | 0.42 | 0.22 | 0.58 | 0.61 | 0.64 | 0.57 | 0.67 | 0.62 | 0.52 | 0.52 | 0.63 | |
| 9 | 0.38 | 0.16 | 0.07 | 0.31 | 0.31 | 0.39 | 0.26 | 0.34 | 0.71 | 0.25 | 0.22 | 0.30 | |
| 10 | 0.41 | 0.18 | 0.09 | 0.75 | 0.57 | 0.62 | 0.76 | 0.75 | 0.31 | 0.74 | 0.75 | 0.70 | |
| 11 | 0.32 | 0.36 | 0.44 | 0.41 | 0.68 | 0.49 | 0.46 | 0.73 | 0.46 | 0.42 | 0.74 | 0.39 | |
| 12 | 0.28 | 0.26 | 0.31 | 0.34 | 0.45 | 0.33 | 0.29 | 0.34 | 0.33 | 0.30 | 0.29 | 0.30 | |
| 13 | 0.45 | 0.50 | 0.70 | 0.62 | 0.82 | 0.67 | 0.81 | 0.76 | 0.67 | 1.02 | 0.68 | 0.62 | |
| 14 | 0.46 | 0.34 | 0.54 | 0.70 | 0.82 | 0.63 | 0.63 | 0.89 | 0.64 | 0.67 | 0.82 | 0.72 | |
| 15 | 0.43 | 0.46 | 0.62 | 0.64 | 0.85 | 0.71 | 0.60 | 0.77 | 0.76 | 0.65 | 0.82 | 0.66 | |

Table S2 Coefficient of determination (R²) regarding their closeness to 1 and the response variability of the strains MMBF 58/09, URM 6997/160821, and IB 19/17

|  | MMBF 58/09 | | URM 6997/160821 | | IB 19/17 | |
| --- | --- | --- | --- | --- | --- | --- |
| Time (h) | R^2^ | Variability (%) | R^2^ | Variability (%) | R^2^ | Variability (%) |
| 24 | 0.7996 | 79.96 | 0.7068 | 70.68 | - | - |
| 48 | 0.9272 | 92.72 | 0.8917 | 89.17 | 0.9080 | 90.80 |
| 72 | 0.8821 | 88.21 | 0.8819 | 88.19 | 0.7855 | 78.55 |
| 96 | 0.8167 | 81.67 | 0.8636 | 86.36 | 0.9825 | 98.25 |

Table S3 Results of IAA (mg mL^-1^) for *T. harzianum* MMBF 58/09 and *T. harzianum* IB 19/17

|  | IAA (mg mL^-1^) | | | | | | | |
| --- | --- | --- | --- | --- | --- | --- | --- | --- |
|  | 24 h | 48 h | 72 h | 96 h | 24 h | 48 h | 72 h | 96 h |
| Assay | *T. harzianum* MMBF 58/09 | | | | *T. harzianum* IB19/17 | | | |
| 1 | - | - | - | - | - | - | - | 0.01 |
| 2 | - | - | - | - | - | - | - | - |
| 3 | 0.081 | - | - | - | - | 0.01 | - | 0.03 |
| 4 | - | - | - | - | - | - | - | - |
| 5 | - | - | - | - | - | - | - | - |
| 6 | - | - | - | 0.05 | - | - | - | - |
| 7 | - | - | - | - | - | - | - | - |
| 8 | - | - | 0.25 | 0.21 | 0.02 | - | 0.02 | - |
| 9 | - | - | - | 0.005 | - | - | - | - |
| 10 | - | - | - | - | - | 0.06 | 0.02 | 0.10 |
| 11 | - | - | - | - | - | - | - | - |
| 12 | - | 0.003 | - | - | - | - | - | - |
| 13 | - | 0.51 | 0.76 | 0.80 | - | - | 0.02 | - |
| 14 | - | 0.36 | 0.45 | 0.43 | - | - | - | - |
| 15 | - | 0.12 | 0.40 | - | - | 0.019 | 0.04 | 0.02 |

Table S4 Siderophore quantification, expressed as Siderophore Units (%), for the 24 h, 48 h, 72 h, and 96 h periods for the strains *T. harzianum* MMBF 58/09, *T. asperellum* URM 6997/160821, and *T. harzianum* IB 19/17

|  | Siderophore Units (%) | | | | | | | | | | | |
| --- | --- | --- | --- | --- | --- | --- | --- | --- | --- | --- | --- | --- |
|  | 24 h | | | 48 h | | | 72 h | | | 96 h | | |
| Assay | MMBF 58/09 | URM 6997/160821 | IB 19/17 | MMBF 58/09 | URM 6997/160821 | IB 19/17 | MMBF 58/09 | URM 6997/160821 | IB 19/17 | MMBF 58/09 | URM 6997/160821 | IB 19/17 |
| 1 | 13.0 | - | - | - | - | 75.0 | - | - | - | - | - | - |
| 2 | 49.20 | - | 11.0 | - | - | - | - | - | - | - | - | - |
| 3 | - | - | - | 75.0 | 93.0 | 88.0 | 73.3 | - | - | - | - | - |
| 4 | 68.0 | - | 41.0 | 98.0 | 94.9 | - | - | 32.2 | - | - | - | - |
| 5 | 67.30 | - | 16.0 | - | 99.4 | 90.2 | - | 13.4 | - | - | - | - |
| 6 | - | 36.45 | - | 88.7 | - | - | - | - | - | - | - | - |
| 7 | 26.0 | - | - | 83.2 | 83.0 | 92.0 | - | - | - | 0.40 | - | - |
| 8 | - | - | 30.0 | - | 59.0 | 90.4 | 6.00 | - | - | - | - | - |
| 9 | 6.30 | - | 37.0 | - | - | - | - | - | - | - | - | - |
| 10 | - | - | - | 84.0 | - | - | - | - | - | 21.1 | - | - |
| 11 | 49.1 | - | 70.3 | - | 86.0 | 97.0 | - | 4.50 | - | 33.26 | - | - |
| 12 | 8.00 | - | 20.2 | 94.0 | - | - | 4.30 | 0.51 | 96.0 | 22.0 | - | - |
| 13 | 59.10 | - | 1.01 | - | - | - | - | 30.0 | - | - | 0.51 | - |
| 14 | 33.04 | - | - | - | - | 92.0 | - | - | - | - | - | - |
| 15 | - | - | - | - | 97.0 | 96.0 | - | - | - | - | - | - |

Table S5 Estimated effect of the Plackett-Burman statistical design showing significant variable effects on siderophore production

| **Variables** | **Estimated Effect** | **Standard Error** | **t** | **p** |
| --- | --- | --- | --- | --- |
| MMBF 58/09 | | | | |
| 96h |  | | | |
| Protein hydrolysate | -12.6600 | 4.498316 | -2.81439 | 0.020232 |
| Inorganic nitrogen | -11.5504 | 4.449152 | -2.59610 | 0.028923 |

Table S6 Chitinase quantification (U mL^-1^) during 24 h, 48 h, 72 h, and 96 h for the strains *T. harzianum* MMBF 58/09, *T. asperellum* URM 6997/160821, and *T. harzianum* IB 19/17

|  | Chitinase (U mL^-1^) | | | | | | | | | | | |
| --- | --- | --- | --- | --- | --- | --- | --- | --- | --- | --- | --- | --- |
|  | 24 h | | | 48 h | | | 72 h | | | 96 h | | |
| Assay | MMBF 58/09 | URM 6997/160821 | IB 19/17 | MMBF 58/09 | URM 6997/160821 | IB 19/17 | MMBF 58/09 | URM 6997/160821 | IB 19/17 | MMBF 58/09 | URM 6997/160821 | IB 19/17 |
| 1 | 0.015 | 0.001 | 0.006 | - | 0.002 | 0.002 | - | 0.004 | 0.004 | - | 0.002 | - |
| 2 | 0.009 | 0.002 | 0.001 | 0.005 | - | 0.005 | - | 0.006 | 0.003 | - | 0.003 | 0.007 |
| 3 | 0.010 | - | 0.002 | - | - | 0.002 | - | - | 0.002 | - | - | 0.006 |
| 4 | 0.015 | 0.005 | 0.0004 | - | - | 0.0005 | - | - | 0.002 | - | - | 0.009 |
| 5 | 0.012 | 0.004 | 0.001 | - | 0.002 | 0.002 | - | - | 0.003 | - | - | 0.003 |
| 6 | 0.022 | 0.002 | 0.003 | - | - | 0.003 | - | 0.002 | 0.003 | - | 0.002 | 0.003 |
| 7 | 0.006 | 0.002 | 0.003 | - | - | 0.002 | - | 0.002 | 0.002 | - | 0.002 | 0.002 |
| 8 | - | 0.002 | 0.002 | - | 0.001 | 0.002 | - | 0.001 | 0.002 | - | 0.001 | 0.001 |
| 9 | - | 0.002 | 0.003 | - | 0.001 | 0.002 | - | 0.002 | 0.001 | - | 0.002 | 0.005 |
| 10 | 0.006 | 0.005 | 0.001 | - | 0.002 | 0.002 | - | 0.001 | 0.002 | - | 0.001 | 0.002 |
| 11 | 0.004 | 0.002 | 0.003 | - | 0.00007 | 0.001 | 0.015 | 0.002 | - | - | 0.002 | 0.004 |
| 12 | - | 0.002 | 0.003 | - | - | 0.0002 | - | 0.0008 | 0.001 | - | 0.0008 | 0.001 |
| 13 | 0.003 | 0.005 | 0.003 | - | 0.002 | 0.002 | - | 0.002 | 0.002 | - | 0.002 | 0.002 |
| 14 | 0.004 | 0.005 | 0.005 | - | 0.002 | 0.002 | - | 0.002 | 0.002 | - | 0.002 | 0.002 |
| 15 | 0.002 | 0.001 | 0.002 | - | 0.001 | 0.001 | - | 0.001 | 0.001 | - | 0.001 | 0.003 |

Table S7 Estimated effect of the statistical analysis of the Plackett-Burman design showing significant values of different variables for chitinase production

| **Variables** | **Estimated Effect** | **Standard Error** | **t** | **p** |
| --- | --- | --- | --- | --- |
| MMBF 58/09 | | | | |
| 24 h |  | | | |
| Glucose | 0.009833 | 0.002142 | 4.590524 | 0.001308 |
| Protein hydrolysate | 0.006167 | 0.002142 | 2.878803 | 0.018214 |
| IB 19/17 | | | | |
| 72 h |  | | | |
| Glucose | 0.001500 | 0.000424 | 3.53733 | 0.006341 |

Table S8 Results of β-1,3-glucanase quantification in U mL^-1^ during the times of 24 h, 48 h, 72 h, and 96 h for the strains *T. harzianum* MMBF 58/09, *T. asperellum* URM 6997/160821, and *T. harzianum* IB 19/17

|  | β-1,3-glucanase ( U mL^-1^) | | | | | | | | | | | |
| --- | --- | --- | --- | --- | --- | --- | --- | --- | --- | --- | --- | --- |
|  | 24 h | | | 48 h | | | 72 h | | | 96 h | | |
| Assay | MMBF 58/09 | URM 6997/160821 | IB 19/17 | MMBF 58/09 | URM 6997/160821 | IB 19/17 | MMBF 58/09 | URM 6997/160821 | IB 19/17 | MMBF 58/09 | URM 6997/160821 | IB 19/17 |
| 1 | 0.004 | 0.081 | 0.012 | - | - | 0.003 | - | - | 0.003 | 0.005 | - | 0.001 |
| 2 | 0.002 | 0.199 | 0.009 | 0.006 | - | 0.003 | - | - | 0.002 | - | - | 0.005 |
| 3 | 0.006 | 0.037 | 0.007 | 0.005 | - | 0.004 | - | - | 0.003 | - | - | 0.008 |
| 4 | 0.005 | 0.040 | 0.002 | - | - | 0.003 | - | - | 0.002 | - | - | 0.004 |
| 5 | 0.003 | 0.023 | 0.002 | 0.005 | - | 0.002 | - | - | 0.003 | - | - | 0.005 |
| 6 | 0.006 | 0.075 | 0.005 | - | - | 0.003 | - | - | 0.003 | - | - | 0.006 |
| 7 | 0.005 | 0.098 | 0.003 | - | - | 0.002 | - | - | 0.003 | - | 0.0009 | 0.008 |
| 8 | 0.006 | 0.048 | 0.001 | - | - | 0.003 | 0.006 | - | 0.003 | 0.006 | - | 0.004 |
| 9 | 0.007 | - | 0.0001 | - | - | 0.004 | 0.006 | - | 0.005 | 0.007 | - | 0.009 |
| 10 | 0.007 | - | 0.002 | 0.004 | - | - | - | - | 0.003 | - | - | 0.006 |
| 11 | 0.005 | 0.089 | 0.00002 | 0.005 | - | 0.002 | 0.004 | - | 0.004 | - | - | 0.007 |
| 12 | 0.005 | 0.063 | 0.003 | - | - | 0.004 | - | - | 0.002 | - | - | 0.008 |
| 13 | 0.005 | 0.075 | 0.004 | - | - | 0.003 | - | - | 0.003 | - | 0.003 | 0.011 |
| 14 | 0.004 | 0.068 | 0.003 | - | - | 0.003 | - | - | 0.004 | - | 0.004 | 0.006 |
| 15 | 0.004 | 0.079 | 0.002 | - | - | 0.002 | - | - | 0.004 | - | 0.002 | 0.009 |

Table S9 Estimated effect of the statistical analysis from the Plackett-Burman design showing significant values of different variables for β-1,3-glucanase production

| **Variables** | **Estimated Effect** | **Standard Error** | **t** | **p** |
| --- | --- | --- | --- | --- |
| MMBF 58/09 | | | | |
| 24 h |  | | | |
| pH | 0.001500 | 0.000599 | 2.50407 | 0.033636 |
| 48 h |  | | | |
| Sucrose | 0.002833 | 0.001210 | 2.34186 | 0.043886 |
| 72 h |  | | | |
| Glucose | -0.002667 | 0.001070 | -2.49277 | 0.034266 |
| 96 h |  | | | |
| Sucrose | -0.003000 | 0.001255 | -2.39116 | 0.040480 |
| URM 6997/160821 | | | | |
| 24 h |  | | | |
| Sucrose | 0.048167 | 0.021032 | 2.29016 | 0.047762 |
| pH | -0.064500 | 0.021032 | -3.06676 | 0.013424 |

Table S10 Results of protease quantification (mg mL^-1^) at 24 h, 48 h, 72 h, and 96 h for the strains *T. harzianum* MMBF 58/09, *T. asperellum* URM 6997/160821, and *T. harzianum* IB 19/17

|  | Protease (mg mL^-1^) | | | | | | | | | | | |
| --- | --- | --- | --- | --- | --- | --- | --- | --- | --- | --- | --- | --- |
|  | 24 h | | | 48 h | | | 72 h | | | 96 h | | |
| Assay | MMBF 58/09 | URM 6997/160821 | IB 19/17 | MMBF 58/09 | URM 6997/160821 | IB 19/17 | MMBF 58/09 | URM 6997/160821 | IB 19/17 | MMBF 58/09 | URM 6997/160821 | IB 19/17 |
| 1 | 1.23 | 1.18 | 0.93 | 0.68 | 0.47 | 0.67 | 0.54 | 0.47 | 0.47 | 0.39 | 0.94 | 0.74 |
| 2 | 0.45 | - | - | 0.20 | 0.21 | 0.14 | 0.38 | 0.21 | 0.09 | - | 0.52 | 0.35 |
| 3 | 0.89 | 0.18 | 1.45 | 1.07 | 0.18 | 0.75 | 0.60 | 0.18 | 0.67 | 0.53 | 0.28 | 0.98 |
| 4 | 1.12 | 0.10 | 1.18 | 1.04 | 0.15 | - | 0.97 | 0.15 | 0.63 | 0.92 | 0.10 | 0.90 |
| 5 | 0.44 | 0.09 | 0.35 | 0.58 | 0.11 | 0.44 | 0.56 | 0.11 | 0.43 | 0.64 | 0.06 | 0.32 |
| 6 | 1.33 | 1.01 | 0.89 | 1.02 | 0.67 | 1.40 | 0.81 | 0.67 | 1.15 | 0.89 | 1.34 | 1.47 |
| 7 | 1.14 | 0.55 | 1.14 | 0.92 | 0.85 | 0.94 | 0.76 | 0.85 | 0.90 | 0.83 | 0.74 | 0.48 |
| 8 | 1.25 | 0.86 | 0.63 | 0.64 | 0.49 | 0.98 | 0.62 | 0.49 | 0.66 | 0.07 | 1.34 | - |
| 9 | 0.06 | 0.22 | - | 0.19 | 0.09 | - | 0.06 | 0.09 | - | - | 0.49 | - |
| 10 | 0.54 | 0.24 | - | 0.55 | 0.22 | 0.39 | 0.30 | 0.22 | 0.61 | 0.27 | 0.95 | 1.29 |
| 11 | 0.17 | - | - | - | 0.16 | 0.20 | 0.03 | 0.16 | 0.17 | 0.10 | 0.55 | 0.81 |
| 12 | 0.16 | - | - | - | - | 0.90 | - | - | - | - | 0.34 | 0.09 |
| 13 | 0.72 | 0.25 | 0.32 | 0.50 | 0.19 | 0.13 | 0.26 | 0.19 | 0.32 | 0.24 | 0.75 | 0.36 |
| 14 | 0.63 | 0.32 | 0.33 | 0.48 | 0.11 | 0.14 | 0.39 | 0.11 | 0.34 | 0.22 | 0.77 | 0.40 |
| 15 | 0.66 | 0.26 | 0.36 | 0.52 | 0.15 | 0.32 | 0.48 | 0.15 | 0.94 | 0.52 | 0.74 | 0.45 |

Table S11 Estimated effect of the statistical analysis of the Plackett-Burman design showing the significant values of different variables for protease production

| **Variables** | **Estimated Effect** | **Standard Error** | **t** | **p** |
| --- | --- | --- | --- | --- |
| MMBF 58/09 | | | | |
| 24 h |  | | | |
| Glucose | 0.240000 | 0.072785 | 3.29737 | 0.009269 |
| Protein hydrolysate | 0.856667 | 0.072785 | 11.76978 | 0.000001 |
| 48 h |  | | | |
| Glucose | 0.208333 | 0.090960 | 2.29037 | 0.047746 |
| Protein hydrolysate | 0.641667 | 0.090960 | 7.05435 | 0.000060 |
| 72 h |  | | | |
| Glucose | 0.248333 | 0.060442 | 4.10865 | 0.002642 |
| Protein hydrolysate | 0.495000 | 0.060442 | 8.18972 | 0.000018 |
| Inorganic nitrogen | 0.186522 | 0.059781 | 3.12008 | 0.012317 |
| 96 h |  | | | |
| Protein hydrolysate | 0.436667 | 0.147569 | 2.959065 | 0.015984 |
| URM 6997/160821 | | | | |
| 24 h |  | | | |
| Protein hydrolysate | 0.555000 | 0.180507 | 3.074675 | 0.013254 |
| 48 h |  | | | |
| Protein hydrolysate | 0.336667 | 0.122543 | 2.747339 | 0.022575 |
| 72 h |  | | | |
| Protein hydrolysate | 0.336667 | 0.122543 | 2.747339 | 0.022575 |
| IB 19/17 | | | | |
| 24h |  | | | |
| Protein hydrolysate | 0.978333 | 0.136886 | 7.147049 | 0.000054 |
| 72h |  | | | |
| Protein hydrolysate | 0.530000 | 0.141571 | 3.743707 | 0.004599 |
| 96h |  | | | |
| Glucose | 0.451667 | 0.183477 | 2.46171 | 0.036058 |
| Inorganic nitrogen | -0.514783 | 0.181472 | -2.83671 | 0.019508 |
